# The Effects of Research Activities on Biomedical Students' Mental Health: A National Cross-Sectional Study

**DOI:** 10.3389/fpsyt.2022.796697

**Published:** 2022-03-29

**Authors:** Yue Li, Shengyang Jin, Ya Li, Fei Guo, Ting Luo, Bo Pan, Mingxing Lei, Yaosheng Liu

**Affiliations:** ^1^Department of Oncology, The Fifth Medical Center of Chinese PLA General Hospital, Beijing, China; ^2^Department of Plastic Surgery, Plastic Surgery Hospital, Chinese Academy of Medical Sciences and Peking Union Medical College, Beijing, China; ^3^Department of Dermatology, Ningxia Yiyang Geriatric Hospital, Yinchuan, China; ^4^Department of Anesthesiology, Second Affiliated Hospital of Air Force Medical University, Xi'an, China; ^5^Department of Obstetrics and Gynecology, Second Xiangya Hospital of Central South University, Changsha, China; ^6^Chinese PLA Medical School, Beijing, China; ^7^Department of Orthopedic Surgery, Hainan Hospital of Chinese PLA General Hospital, Sanya, China; ^8^Department of Orthopedic Surgery, The Fourth Medical Center of Chinese PLA General Hospital, Beijing, China; ^9^National Clinical Research Center for Orthopedics, Sports Medicine & Rehabilitation, Chinese PLA General Hospital, Beijing, China

**Keywords:** mental health, cross-sectional study, anxiety, depression, research work duration, biomedical students

## Abstract

Mental health disorders are prevalent among biomedical students, and scientific research is one of their main activities, yet less is known about the relationship between research activities and mental health among these students. The aim of this cross-sectional study was to assess the associations between research activities and mental health and to identify the potential risk factors for anxiety and depression among biomedical students in China. This study enrolled 1,079 participants between November 2020 and December 2020 from 29 Chinese provinces and collected participants' basic characteristics, work situations, scientific achievements, and potential stress sources *via* an online questionnaire. Anxiety and depression were evaluated by two widely used scales, the Generalized Anxiety Disorder-7 (GAD-7) and the Patient Health Questionnaire-9 (PHQ-9), respectively. The study also assessed the associations between scientific research duration and mental health. Univariate and multivariate analyses were performed to identify the predictors of anxiety and depression. Among the participants, 39.02% scored as having moderate to severe anxiety, and 37.54% scored as having moderate to severe depression. When the Youden index reached its maximum, the optimal cutoff was 7.17 h for the GAD-7 and 6.83 h for the PHQ-9. After adjustment for confounders, a longer research work duration was significantly associated with a higher anxiety [odds ratio (OR) = 1.32, 95% confidence interval (CI): 1.21–1.44, *p* < 0.01] and depression (OR = 1.27, 95% CI: 1.17–1.39, *p* < 0.01). Of all the participants working for 7 h a day, 37.04% had already published at least one paper and 25.93% had at least one Science Citation Index paper. Anxiety and depression are common among biomedical students. The research work duration of 7 h a day is the best cutoff for mental health, and it is associated with acceptable scientific research achievements. Not more than 7 h a day on research is recommended for biomedical students to maintain a balance between mental health and scientific research achievements.

## Introduction

Mental health disorders are a persistent problem among medical students. According to the research literature, more than 30% of the medical students worldwide suffer from depression or anxiety (1, 2). Mental health disorders can lead to personality distortion, poor professional performance and personal relationships, and suicidal tendencies (1, 3–7). Many biomedical students, especially in China, are obliged to engage simultaneously in scientific research and attend a clinical residency training program to meet the qualifications for graduation and becoming a doctor. The heavy clinical training program and scientific research workloads might increase the risk of job burnout (8) and subsequently compromise psychological stability.

Various factors could contribute to anxiety and depression among medical students, including uncertainties about prospects, academic pressures, financial hardships, personal relationships, and lifestyles (9). Among the above factors, academic pressure is an important source of mental disorders (4). In addition, a large number of pressures force the biomedical students to devote time to scientific research for more achievements, potentially aggravating students' mental health disorders. Therefore, an investigation of the relationship between academic activities and mental health is urgently needed. A few studies have focused on the pressure of research, analyzing the impact of research work duration and other potential risk factors on mental health.

Therefore, the aims of this study were to investigate the academic pressure status, elucidate the relationship between research duration and mental health, and investigate potential contributors to scientific research pressure among biomedical students. We hypothesized that academic pressure was strong among biomedical students and that the research work time played a role in affecting mental health.

## Materials and Methods

### Participants and Study Design

This study prospectively collected 1,079 biomedical students between November 2020 and December 2020 from 29 provinces in China. We distributed a questionnaire online *via* the commonly used communication tools, such as the WeChat software, emails, and telephone messages. The non-probability snowball sampling strategy (10) was used to recruit biomedical students from all over China. The target participants were medical postgraduate and doctoral students with good academic performance, such as students studying in the Xiangya School of Medicine (Changsha, Hunan Province), the Peking Union Medical College (Beijing), and the West China Medical Center (Xi'an, Shaanxi Province). The students were invited to share the survey link or the quick response code with the classmates who were willing to participate in the study. All participants confirmed their understanding of the informed consent form and volunteered to participate in the survey. Participants' basic characteristics, work situations, research achievements, and potential stress sources were collected *via* an online questionnaire. This online questionnaire was conducted anonymously, and no identifiable personal information was collected. The questionnaire included approximately 20 questions, and it took 3–5 min to complete. After a month, we ended the online survey distribution, having collected sufficient samples for the analysis. The study protocol complied with the Declaration of Helsinki. It was approved by the ethics committee of the Plastic Surgery Hospital (Institute), the Chinese Academy of Medical Sciences (CAMS), and the Peking Union Medical College (no. 2020–157). It was registered at www.chictr.org.cn (no. ChiCTR2000039574). A signed informed consent was obtained from each participant after the aim of the study had been explained to them and after they had been told that they had the right to withdraw at any time if they were unwilling to continue with the investigation. The study adhered to the Strengthening the Reporting of Observational Studies in Epidemiology (STROBE) Statement (11).

### Sample Size

In the error rates, 1 – beta was set to 0.90, and alpha was 0.05. The numbers for independent controlled and tested variables were each 20, and *R*^2^ was 0.05. Applying a multiple regression formula for a cross-sectional survey resulted in a recommended sample size of 487. The non-response rate was 10%, and the final sample size was 536. The sample size was calculated using the Power Analysis and Sample Size (PASS) software 11.0.10.

### Evaluation of Research Work Duration and Mental Health

The research work duration (hours) refers to the time spent on scientific research per day, mainly including basic academic experiments and clinical investigations, such as study design, literature review, and data collection. In the mental health area, anxiety and depression were evaluated. Anxiety was assessed by the Patient Generalized Anxiety Disorder-7 (GAD-7) (12), and depression was assessed by the Patient Health Questionnaire-9 (PHQ-9) (13). The GAD-7 with scores ranging from 0 to 21 is widely used to evaluate the anxiety status. The severity of anxiety was classified as follows: minimal (0–4), mild (5–9), moderate (10–14), and severe (15 or above). The classification of the PHQ-9 (ranging from 0 to 27) was consistent with GAD-7. Both scales are authoritative, commonly used, and validated in the previous studies (14–18). Subgroup analyses were performed on the research work duration and mental health. Subgroup analyses were also performed on research work duration and scientific achievements. In this study, scientific achievements were evaluated by the number of published scientific papers in Chinese or English as the first or co-first authors, which means that the participants made the major contribution to the published paper.

Sensitivity and specificity were calculated and evaluated to explore statistically and confirm the best cutoff value of the research work duration for mental health status. The corresponding optimal cutoff value was obtained when the Youden index reached the maximum. The Youden index: (sensitivity + specificity) – 1. Binary variables were used to calculate sensitivity and specificity. A number of three binary variables were developed for GAD-7. The first was participants with minimal anxiety (GAD-7 ≤ 4) vs. participants with mild and above mild anxiety (GAD-7 ≥ 5). The second was participants with minimal and mild anxiety (GAD 7 ≤ 9) vs. participants with moderate and severe anxiety (GAD 7 ≥ 10). The third was participants with moderate or below moderate anxiety (GAD-7 ≤ 14) vs. participants with severe anxiety (GAD-7 ≥ 15). The best cutoff for the research work duration for GAD-7 was the mean value of the above three cutoffs. The corresponding three binary variables were developed for the PHQ-9, and the optimal cutoff value was evaluated.

### Definitions of Characteristics

The biomedical students in the study indicate that they are studying in medical schools to obtain their master's degrees, PhDs, or postdocs, and plenty of them are students before becoming doctors. Some medical schools are located in economically developed first-class cities, including Beijing, Shanghai, Guangzhou, and Shenzhen (9). The “double first-class” university strategic plan refers to the nation-supported program to build first-class universities and disciplines worldwide. There are two types of biomedical students in China: academic medical students and clinical medical students (19). Clinical medical students are required to take the clinical standardized residency training program for at least 3 years and are more likely to do clinical research, while academic medical students are more likely to do basic science research. There are no requirements for them to take a clinical residency training program. Participants' mentor with an administrative post includes department directors, deputy department directors, hospital directors, and deputy hospital directors. Participants' mentor with an academic post includes the positions in the Chinese Medical Association and corresponding provincial medical associations. The National Natural Science Foundation of China (NSFC) was established in 1986 and has attracted many scholars to contribute to science and technology (20).

### Potential Risk Factors for Mental Health

Univariate and multivariate analyses were performed to identify the potential risk predictors for anxiety and depression. We collected and analyzed 20 potential risk factors, including participants' basic characteristics [age: (<25 years vs. ≥25 and <30 years vs. ≥30], grade (first vs. second vs. third vs. fourth year vs. deferment), degree course (master's degree vs. PhD vs. postdoctoral), department (basic medicine vs. internal medicine vs. surgery vs. stomatology vs. gynecology and obstetrics vs. ophthalmology vs. anesthesiology vs. others), professional type (academic vs. clinical), marital status (single vs. dating vs. married without children vs. married with children), number of children (0 vs. 1 vs. ≥ 2), disposable income [Renminbi (RMB), per month] (<1,000 vs. ≥ 1,000 and ≥ 3,000 vs. ≥ 3,000 and <5,000 vs. ≥ 5,000), university located in a first-class city (yes vs. no), and university involvement in the national “double first-class” strategic plan (yes vs. no), variables relating to research activities (research type (basic research vs. clinical research vs. both vs. uncertain), duration of research work (hours, per day) (0–2 vs. 3–5 vs. 6–8 vs. 9–11 vs. ≥12), number of participated research projects (0 vs. 1 vs. 2 vs. 3 vs. ≥ 4), participant's mentor having an administrative post (yes vs. no), participant's mentor having an academic post (yes vs. no), participant's mentor having projects funded by the NSFC (yes vs. no), participants having research directors (yes vs. no), participant's research achievements (number of published papers (0 vs. 1 vs. 2 vs. 3 vs. 4 vs. ≥ 5), total impact factors of published papers (0 vs. > 0 and ≤ 3 vs. > 3 and ≤ 6 vs. > 6 and ≤ 10 vs. > 10), and participants' previous mental health status (previous clinical diagnosis of anxiety or depression (yes vs. no).

### Statistical Analysis

The scores for the GAD-7 and the PHQ-9 were presented as mean ± standard deviation (SD). The subgroup analysis of continuous variables was performed using the one-way analysis of variance (ANOVA) and non-parametric or mixed tests. The subgroup analysis of counted variables between different research time groups was conducted using the chi-square test. The participants with no research work (the research work time was 0 h) were taken as the control group. A comparison between the control group and the study group (participants with a research work time of 1 or more hours) was conducted using the Wilcoxon two-sample test. Violin plots were made to visualize the corresponding data. Receiver operation curves (ROC) were drawn for anxiety and depression, and the area under the curve (AUC) was calculated to evaluate the performance. Data were analyzed using SAS 9.4 for Windows (SAS Institute Inc., Cary, NC, United States). All tests were two-tailed, with a significance level of 5%.

## Results

### Participants' Basic Characteristics

A number of 1,079 valid responses were received. The majority of the participants (75.07%, 810/1,079) held a masters' degree, followed by PhD students (23.82%, 257/1,079), and postdoctoral students (1.11%, 12/1,079). The proportions of academic and clinical medical students were similar [53.75% (580/1,079) vs. 46.25% (499/1,079)]. The proportion of the participants who spent no more than 5 h a day on research was 48% (518/1,079), and 52% (556/1,079) of the participants spent 6 or more hours a day on research. A majority of the students (62.84%, 678/1,079) also reported that they had a research director or assistant. However, the proportion of the participants who reported that they did research mainly by self-learning was relatively high at 37.16% (401/1,079).

**Figure 1:**
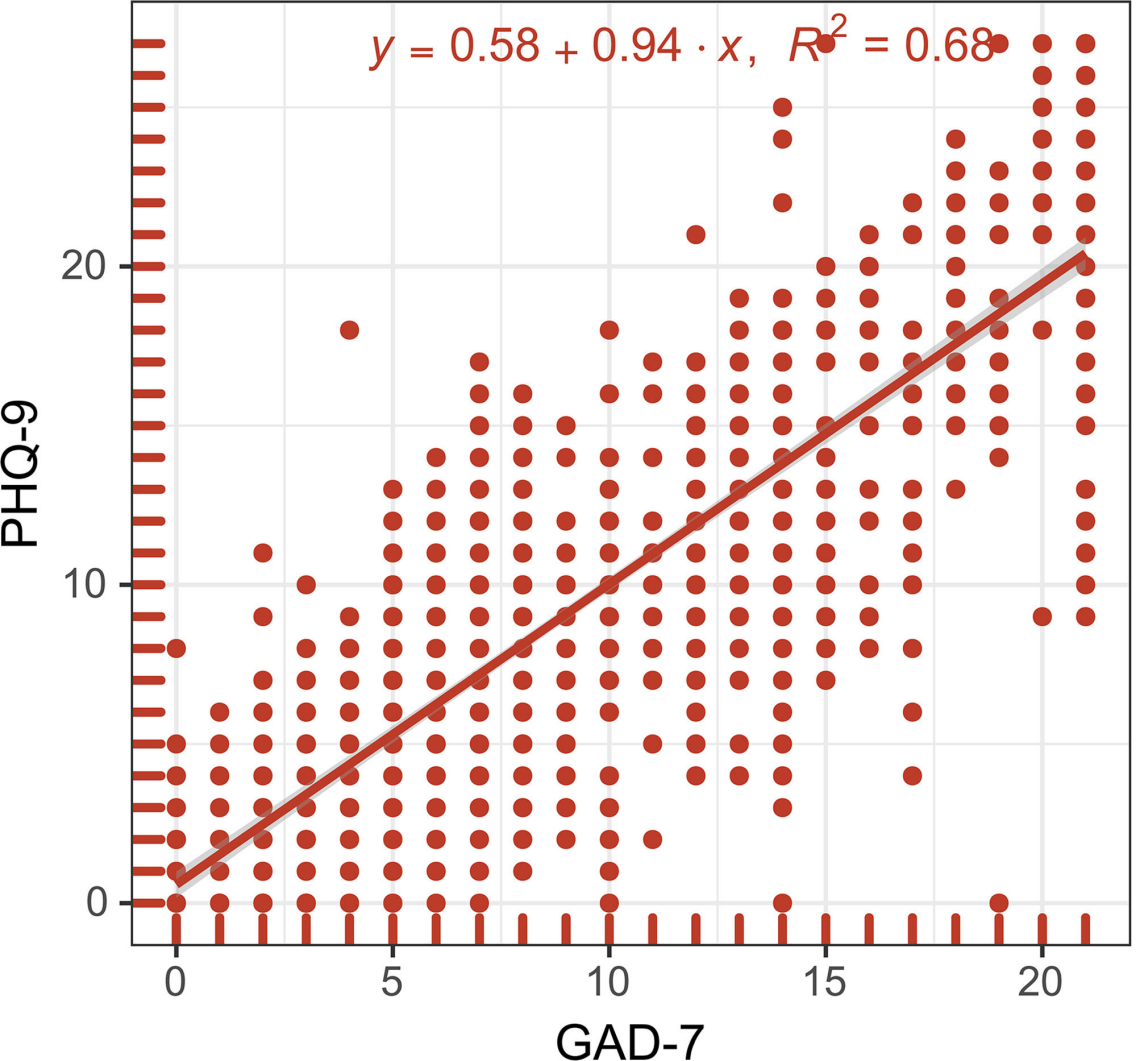
Linear plot of GAD-7 against PHQ-9. A smooth red line was fitted using the linear regression model: PHQ-9 = 0.94*GAD-7 + 0.58, R^2^ = 0.68, *p* < 0.0001. The gray shadow around the red line indicates 95% confidence interval. GAD-7 indicates Generalized Anxiety Disorder-7; PHQ-9 indicates Patient Health Questionnaire-9; *x* indicates GAD-7; and *y* indicates PHQ-9.

Concerning anxiety and depression, the scores for 39.02% (421/1,079) of the biomedical students indicated moderate to severe anxiety, and 37.54% (405/1,079) had scores indicating moderate to severe depression. Table 1 shows the additional details of the participants' general characteristics, research activities, and scientific achievements. We also found that the anxiety and depression scores were significantly associated, and a linear equation was developed: PHQ-9 = 0.94^*^GAD-7 + 0.58 (R^2^ = 0.68, *p* < 0.0001, Figure 1).

**Table 1:** Participant's demographic characteristics.

**Characteristics**	**Number of participants** **(*n* = 1,079)**
**Basic characteristics**	
Age (years)	
<25	46.15% (498/1,079)
≥25 and <30	42.91% (463/1,079)
≥30	10.94% (118/1,079)
**Grade**	
First year	40.04% (432/1,079)
Second year	35.22% (380/1,079)
Third year	21.13% (228/1079)
Fourth year	1.85% (20/1,079)
Deferment	1.76% (19/1,079)
**Pursued degree**	
Master's degree	75.07% (810/1,079)
PhD	23.82% (257/1,079)
Postdoctoral	1.11% (12/1,079)
**Department**	
Basic medicine	6.58% (71/1,079)
Internal medicine	25.58% (276/1,079)
Surgery	20.85% (225/1,079)
Stomatology	3.43% (37/1,079)
Gynecology and obstetrics	5.19% (56/1,079)
Ophthalmology	4.08% (44/1,079)
Anesthesiology	3.89% (42/1,079)
Others	30.40% (328/1,079)
**Professional type**	
Academic	53.75% (580/1,079)
Clinical	46.25% (499/1,079)
**Marital status**	
Single	49.77% (537/1,079)
Dating	36.52% (394/1,079)
Married without children	5.56% (60/1,079)
Married with children	8.16% (88/1,079)
**Number of children**	
0	91.84% (991/1,079)
1	6.77% (73/1,079)
≥2	1.39% (15/1,079)
**Disposable income (RMB, month)**	
<1,000	22.06% (238/1,079)
≥1,000 and <3,000	59.31% (640/1,079)
≥3,000 and <5,000	8.34% (90/1,079)
≥5,000	10.29% (111/1,079)
**University located in a first-class city**	
Yes	22.98% (248/1,079)
No	77.02% (831/1,079)
**University involvement in the national “double first-class” strategic plan**	
Yes	75.81% (818/1,079)
No	24.19% (261/1,079)
**Variables relating to research activities**	
**Research type**	
Basic research	36.14% (390/1,079)
Clinical research	32.53% (351/1,079)
Both	25.21% (272/1,079)
Uncertain	6.12% (66/1,079)
**Duration of research work (hours, per day)**	
0–2	28.54% (308/1,079)
3–5	19.46% (210/1079)
6–8	20.95% (226/1,079)
9–11	16.96% (183/1,079)
≥12	14.09% (152/1,079)
**Number of participated research projects**	
0	26.60% (287/1,079)
1	42.91% (463/1,079)
2	20.02% (216/1,079)
3	7.60% (82/1,079)
≥4	2.87% (31/1,079)
**Participant's mentor having and administrative post**	
Yes	11.86% (128/1,079)
No	88.14% (951/1,079)
**Participant's mentor having an academic post**	
Yes	10.19% (110/1,079)
No	89.81% (969/1,079)
**Participant's mentor having projects funded by the NSFC**	
Yes	69.51% (750/1,079)
No	30.49% (329/1,079)
**Participants having research director**	
Yes	62.84% (678/1,079)
No	37.16% (401/1,079)
**Participant's research achievements**	
**Number of published papers**	
0	67.10% (724/1,079)
1	15.66% (169/1,079)
2	7.14% (77/1,079)
3	4.17% (45/1,079)
4	2.13% (23/1,079)
≥5	3.80% (41/1,079)
**Total impact factors of published SCI papers**	
0	76.74% (828/1,079)
>0 and ≤ 3	8.90% (96/1,079)
>3 and ≤ 6	8.16% (88/1,079)
>6 and ≤ 10	3.43% (37/1,079)
>10	2.78% (30/1,079)
**Mental health status**	
**Previous clinical diagnosis of anxiety or depression**	
Yes	4.91% (53/1,079)
No	95.09% (1,026/1,079)
**Severity of anxiety (the GAD-7 scale)**	
Minimal	21.13% (228/1,079)
Mild	39.85% (430/1,079)
Moderate	22.06% (238/1,079)
Severe	16.96% (183/1,079)
**Severity of depression (the PHQ-9 scale)**	
Minimal	24.84% (268/1,079)
Mild	37.63% (406/1,079)
Moderate	17.98% (194/1,079)
Severe	19.56% (211/1,079)

*Ph.D., Doctor of Philosophy Degree; SCI, Science Citation Index. RMB, Renminbi; NSFC, National Natural Science Foundation of China; GAD-7, Generalized Anxiety Disorder 7; PHQ-9, Patient Health Questionnaire 9*.

### Relationship Between the Research Work Duration and Mental Health

**Figure 2:**
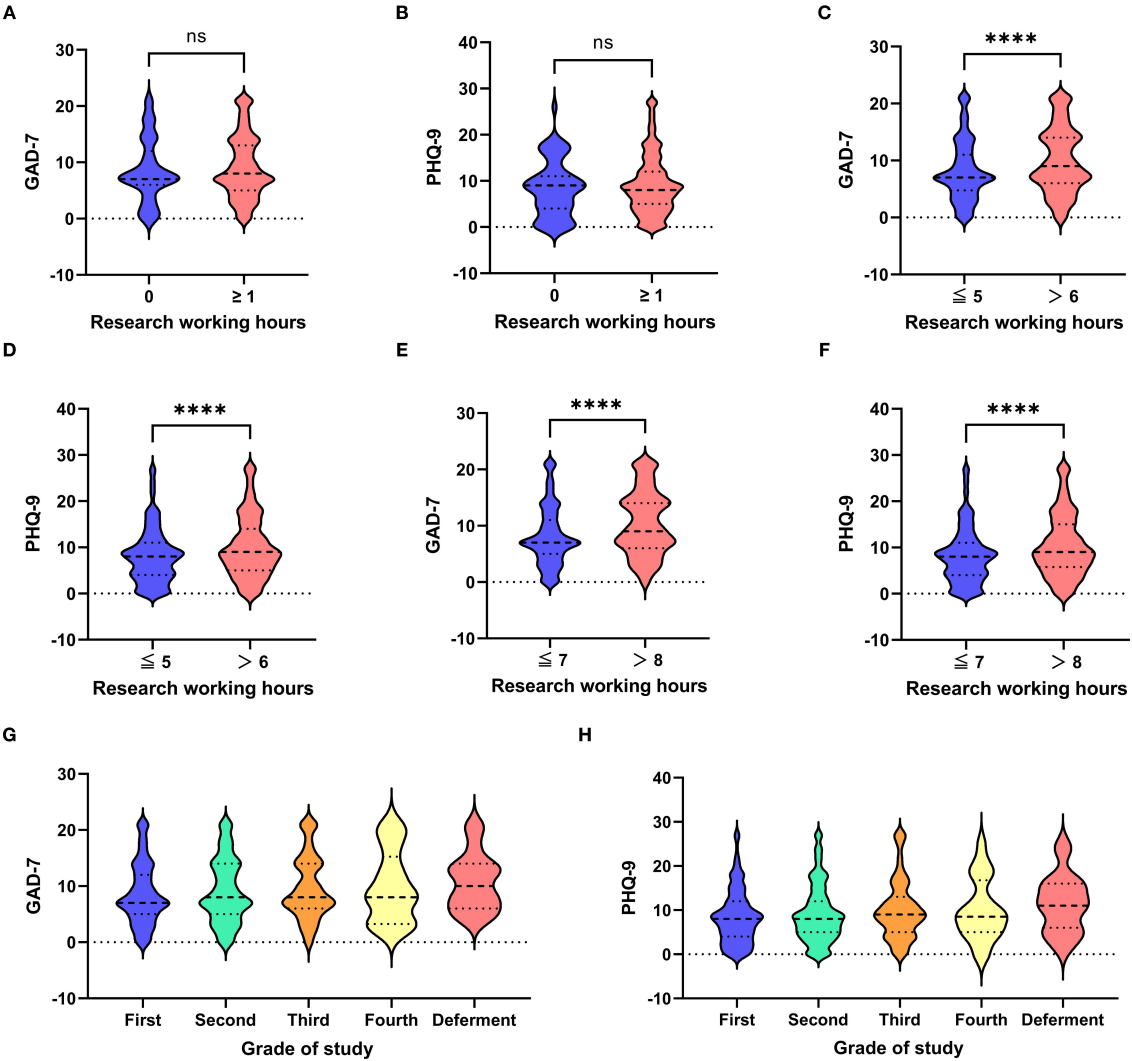
Violin plots for subgroup analysis of research work time and grade of study. Subgroup analysis of GAD-7 between the control group (participants with a research time of 0 h) and the study group (participants with a research time of 1 or more hours) **(A)**. Subgroup analysis of PHQ-9 between the control group (participants with a research time of 0 h) and the study group (participants with a research time of 1 or more hours) **(B)**. Subgroup analysis of GAD-7 between participants with a research time of 5 or fewer hours and participants with a research time of 6 or more hours **(C)**. Subgroup analysis of PHQ-9 between participants with a research time of 5 or fewer hours and participants with a research time of 6 or more hours **(D)**. Subgroup analysis of GAD-7 between participants with a research time of 7 or fewer hours and participants with a research time of 8 or more hours **(E)**. Subgroup analysis of PHQ-9 between participants with a research time of 7 or fewer hours and participants with a research time of 8 or more hours **(F)**. The grade of the study was plotted against GAD-7 **(G)**. The grade of the study was plotted against PHQ-9 **(H)**. GAD-7 indicates Generalized Anxiety Disorder-7; PHQ-9 indicates Patient Health Questionnaire-9. **** indicates *p* < 0.0001; ns indicates no significance.

Participants with no research work (research work time was 0 h) were taken as the control group. A comparison between the control group and the study group (participants with a research time of 1 or more hours) was conducted. The mean GAD-7 was slightly lower in the control group than in the study group (8.51 ± 5.54 vs. 9.10 ± 5.52), but the difference between the two groups was not significant (0.59, 95% CI: −0.81–2.00, *p* = 0.52, Figure 2A). A similar result was observed in PHQ-9 (0.79, 95% CI: −0.82–2.41, *p* = 0.50, Figure 2B).

**Figure 3:**
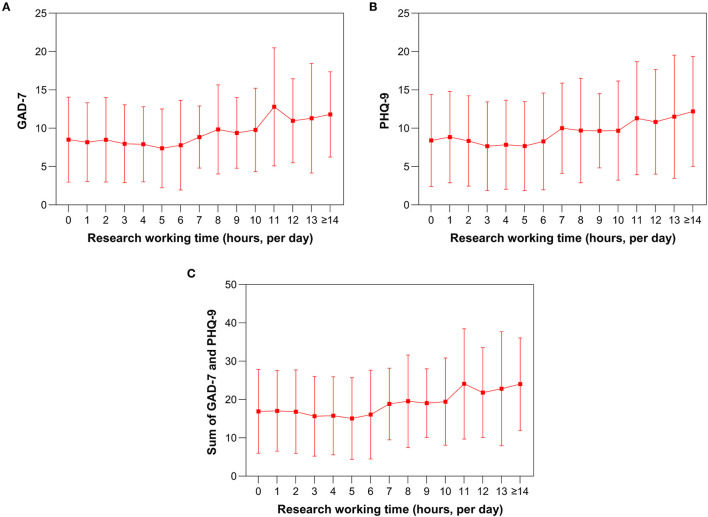
Line chart of research work time (hours) against mental health status. Change in GAD-7 with research work time **(A)**. Change in PHQ-9 with research work time **(B)**. Change in the sum of the GAD-7 and PHQ-9 with research work time **(C)**. GAD-7 indicates Generalized Anxiety Disorder-7; PHQ-9 indicates Patient Health Questionnaire-9.

As the research work time increased, the GAD-7 scores decreased slightly from 8.51 ± 5.54 at <1 h of research work time per day to 7.38 ± 5.14 at 5 h of research work time per day and then increased to 11.80 ± 5.55 at 14 or more hours of research work per day (Figure 3A and Table 2). Similar trends were observed in the PHQ-9 scores (Figure 3B) and in the sum of the GAD-7 and PHQ-9 scores (Figure 3C). The mean GAD-7 was 8.15 ± 5.23 for the participants with a research time of 5 or fewer hours and 9.91 ± 5.65 for participants with a research time of 6 or more hours. The difference between the two groups was 1.76 (95% CI: 1.11–2.42, *p* < 0.0001, Figure 2C). A similar trend was observed in PHQ-9 (1.80, 95% CI: 1.05–2.55, *p* < 0.0001, Figure 2D).

**Table 2:** Research working duration and corresponding anxiety and depression scores.

**Time (hours)**	**Students** **(*n* = 1,079)**	**GAD-7**	**PHQ-9**	**GAD-7 + PHQ-9**
0	63	8.51 ± 5.54	8.40 ± 6.02	16.90 ± 10.91
1	110	8.19 ± 5.13	8.85 ± 5.96	17.02 ± 10.53
2	135	8.48 ± 5.50	8.35 ± 5.90	16.83 ± 10.89
3	90	7.98 ± 5.07	7.66 ± 5.80	15.63 ± 10.41
4	72	7.90 ± 4.90	7.85 ± 5.80	15.75 ± 10.19
5	48	7.38 ± 5.14	7.67 ± 5.80	15.04 ± 10.64
6	80	7.79 ± 5.84	8.29 ± 6.31	16.08 ± 11.59
7	27	8.85 ± 4.04	10.00 ± 5.89	18.85 ± 9.35
8	119	9.85 ± 5.81	9.70 ± 6.82	19.55 ± 12.07
9	41	9.39 ± 4.61	9.66 ± 4.85	19.05 ± 8.95
10	132	9.77 ± 5.44	9.68 ± 6.47	19.45 ± 11.40
11	10	12.80 ± 7.70	11.30 ± 7.38	24.10 ± 14.39
12	77	10.97 ± 5.47	10.83 ± 6.82	21.81 ± 11.70
13	10	11.30 ± 7.15	11.50 ± 8.03	22.80 ± 14.87
≥14	65	11.80 ± 5.55	12.20 ± 7.16	24.00 ± 12.07
P^a^	N.A.	<0.01	<0.01	<0.01

*GAD-7, Generalized Anxiety Disorder 7; PHQ-9, Patient Health Questionnaire 9; N.A., not applicable*.

a *indicates P-values obtained from the one-way ANOVA and nonparametric or mixed test*.

However, taking 5 or fewer hours per day as a cutoff was not optimal. When the sensitivity and specificity were calculated, and the Youden index reached its maximum, the optimal cutoff value was 7.50 h for the first GAD-7 binary variable, 7.50 h for the second GAD-7 binary variable, and 6.50 h for the third GAD-7 binary variable. Therefore, the best cutoff value was 7.17 h (the mean value of the above three best cutoff values) for the general GAD-7. The same three binary variables were developed for PHQ-9. The best cutoff value was 6.50 h for the first PHQ-9 binary variable, 7.50 h for the second PHQ-9 binary variable, and 6.50 h for the third PHQ-9 binary variable. Therefore, the best cutoff value was 6.83 h for the general PHQ-9.

Consequently, the best cutoff value for mental health status was 7.00 h because the mean of 7.17 h (the best cutoff value for the general GAD-7) and 6.83 h (the best cutoff value for the general PHQ-9) is 7.00 h. Violin plots were made to visualize the data. The mean GAD-7 was 8.13 ± 5.26 for participants with a research time of 7 or fewer hours and 10.35 ± 5.62 for participants with a research time of 8 or more hours. The difference between them was 2.22 (95% CI: 1.56–2.87, *p* < 0.0001, Figure 2E). A similar trend was observed in PHQ-9 between the two groups (mean difference: 2.02, 95% CI: 1.26–2.78, *p* < 0.0001, Figure 2F). Figures 3A–C also show that the anxiety and depression scores increased after 7 h per day and the scores remained relatively stable for <7 h per day.

### Relationship Between Research Work Duration and Research Achievements

**Figure 4:**
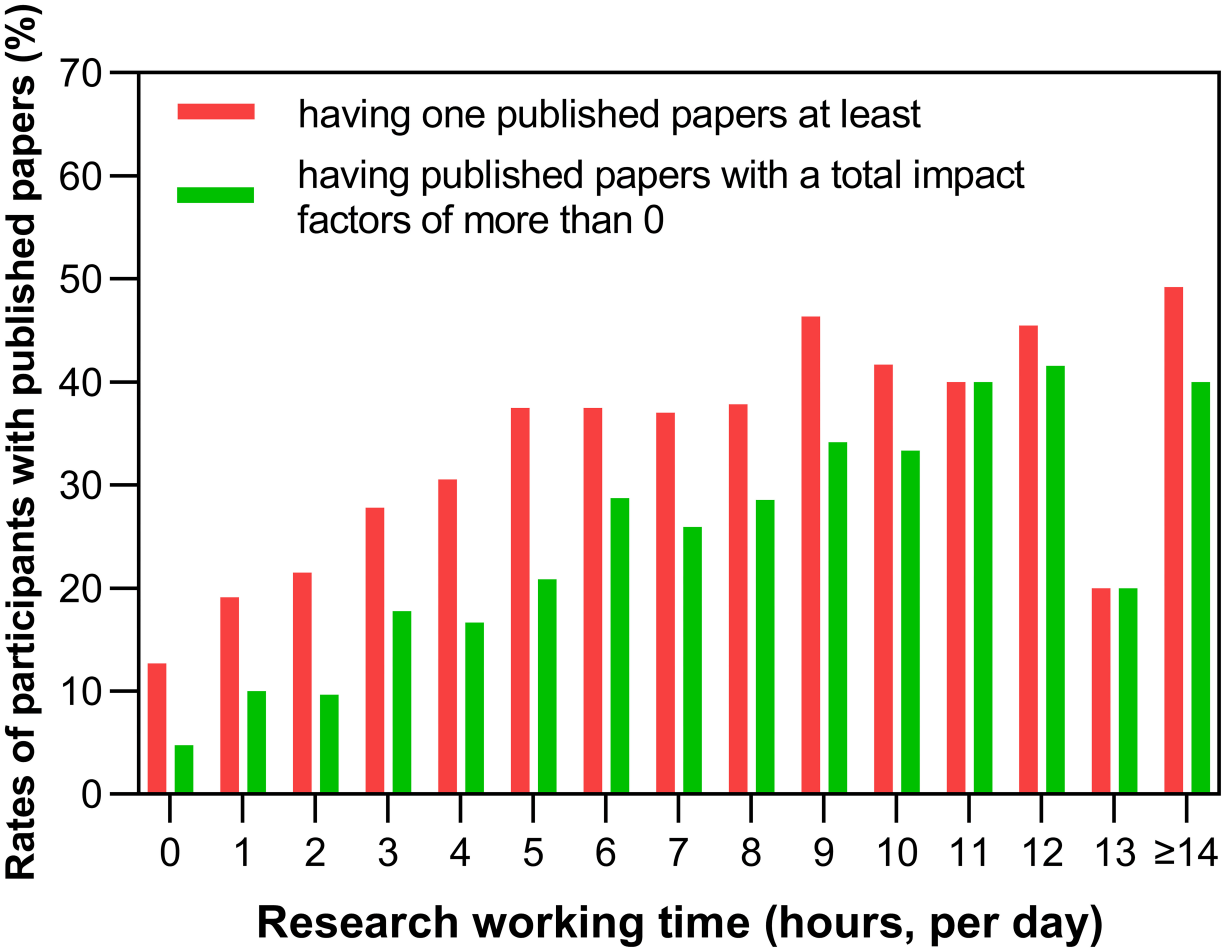
Scientific achievements and research work time. The rates of the participants with published papers were plotted against their research work time (hours per day).

The rates of the participants having at least one published scientific paper increased with extended research work time, and the rate increased from 12.70% at <1 h to 37.04% at 7 h per day (Table 3). For those who worked for more than 7–8 h per day, the rates of having at least one published scientific paper increased slowly (Figure 4). The highest rate of the participants having at least one published scientific paper was 49.23% at 14 or above hours of research work time per day. Supplementary Table 1 shows more details.

**Table 3:** Number of and total impact factor of published papers and corresponding research working time per day.

**Time** **(hours)**	**Students** **(*n* = 1,079)**	**Number of published papers**		**Total impact factors of published papers**			
		**0**	**≥1**	**0**	**>0**	**>3**	**>6**
0	63	87.30% (55/63)	12.70% (8/63)	95.24% (60/63)	4.76% (3/63)	1.59% (1/63)	1.59% (1/63)
1	110	80.91% (89/110)	19.09% (21/110)	90.00% (99/110)	10.00% (11/110)	2.73% (3/110)	0.00% (0/110)
2	135	78.52% (106/135)	21.48% (29/135)	90.37% (122/135)	9.63% (13/135)	2.96% (4/135)	0.74% (1/135)
3	90	72.22% (65/90)	27.78% (25/90)	82.22% (74/90)	17.78% (16/90)	8.89% (8/90)	1.11% (1/90)
4	72	69.44% (50/72)	30.56% (22/72)	83.33% (60/72)	16.67% (12/72)	9.72% (7/72)	5.56% (4/72)
5	48	62.50% (30/48)	37.50% (18/48)	79.17% (38/48)	20.83% (10/48)	10.42% (5/48)	4.17% (2/48)
6	80	62.50% (50/80)	37.50% (30/80)	71.25% (57/80)	28.75% (23/80)	18.75% (15/80)	12.50% (10/80)
7	27	62.96% (17/27)	37.04% (10/27)	74.07% (20/27)	25.93% (7/27)	11.11% (3/27)	11.11% (3/27)
8	119	62.18% (74/119)	37.82% (45/119)	71.43% (85/119)	28.57% (34/119)	16.81% (20/119)	6.72% (8/119)
9	41	53.66% (22/41)	46.34% (19/41)	65.85% (27/41)	34.15% (14/41)	19.51% (8/41)	4.88% (2/41)
10	132	58.33% (77/132)	41.67% (55/132)	66.67% (88/132)	33.33% (44/132)	27.27% (36/132)	12.12% (16/132)
11	10	60.00% (6/10)	40.00% (4/10)	60.00% (6/10)	40.00% (4/10)	40.00% (4/10)	20.00% (2/10)
12	77	54.55% (42/77)	45.45% (35/77)	58.44% (45/77)	41.56% (32/77)	23.38% (18/77)	12.99% (10/77)
13	10	80.00% (8/10)	20.00% (2/10)	80.00% (8/10)	20.00% (2/10)	20.00% (2/10)	10.00% (1/10)
≥14	65	50.77% (33/65)	49.23% (32/65)	60.00% (39/65)	40.00% (26/65)	32.31% (21/65)	9.23% (6/65)
P^a^	N.A.	N.A.	P <0.01^b^	N.A.	P <0.01^c^	P <0.01^c^	P <0.01^c^

*N.A., not applicable*.

a *indicates P-values obtained from the chi-square test*;

b *indicates the number of published papers were statistically different between the 15 research time groups*;

c *indicates the total impact factors of published papers were statistically different between the 15 research time groups*.

The number of participants with at least one SCI paper gradually increased from 4.76% at <1 h of research work time per day to 40.00% for those with 14 or more hours of research work per day. At 7 h of research work per day, the rate of the participants with at least one SCI paper was 25.93%. Supplementary Table 2 contains more details on the total impact factor of published papers for different durations of research work.

### Risk Factors for Predicting Anxiety

Univariate and multivariate analyses of variables for predicting anxiety were performed for the Chinese biomedical students (Table 4). Figures 2G,H show that the anxiety scores and the depression scores increased with the grade of the study, indicating that the higher-grade students suffered from more anxiety and depression due to the pressure experienced in the process of achieving a medical qualification. Based on the multiple logistic regression analysis, clinical medical (OR = 1.40, 95% CI: 1.11–1.78, *p* < 0.01), longer duration of research work (OR = 1.32, 95% CI: 1.21–1.44, *p* = 0.01), no research directors (OR = 1.41, 95% CI: 1.12–1.77, *p* = 0.01), and previous clinical diagnosis of anxiety or depression (OR = 5.38, 95% CI: 3.18–9.11, *p* < 0. 01) were associated with higher anxiety scores, while higher disposable income per month (OR = 0.79, 95% CI: 0.69–0.90, *p* < 0.01) was relevant to lower anxiety scores, indicating that the variable was a protective independent risk factor.

**Table 4:** The univariate and multivariate analysis of variables for predicting anxiety in Chinese medical graduate students.

**Characteristics**	**GAD-7** **(mean ± SD)**	**Single logistic regression**		**Multiple logistic regression**	
		**OR (95% CI)**	***P***	**OR (95% CI)**	***P***
**Age (years)**					
≥25	8.90 ± 5.45	1.09 (0.92–1.28)	0.33	Not entered into the model	
≥25 and <30	9.16 ± 5.66			
≥30	9.44 ± 5.28			
**Pursued degree**					
Master's degree	9.01 ± 5.56	1.10 (0.87–1.39)	0.41	Not entered into the model	
PhD	9.23 ± 5.38			
Postdoctoral	9.75 ± 6.27			
**Grade**					
First year	8.58 ± 5.37	1.16 (1.03–1.31)	0.01	Not entered into the model	
Second year	9.11 ± 5.63			
Third year	9.71 ± 5.54			
Fourth year	9.85 ± 6.31			
Deferment	10.68 ± 5.09			
**Department**					
Basic medicine	8.45 ± 5.40	0.96 (0.92–1.00)	0.06	Not entered into the model	
Internal medicine	9.54 ± 5.55			
Surgery	9.62 ± 5.78			
Stomatology	8.43 ± 4.52			
Gynecology and obstetrics	9.93 ± 5.60			
Ophthalmology	7.84 ± 5.72			
Anesthesiology	8.89 ± 5.44			
Others	8.54 ± 5.36			
**Professional type**					
Academic	8.97 ± 5.50	1.06 (0.85–1.32)	0.60	1.40 (1.11–1.78)	<0.01
Clinical	9.18 ± 5.55				
**Marital status**					
Single	8.75 ± 5.59	1.08 (0.96–1.22)	0.19	Not entered into the model	
Dating	9.48 ± 5.51			
Married without children	9.38 ± 5.72			
Married with children	8.92 ± 4.97			
**Number of children**					
0	9.08 ± 5.57	1.03 (0.75–1.42)	0.85	Not entered into the model	
1	8.66 ± 5.09			
≥2	10.20 ± 4.26			
**Disposable income (RMB, month)**					
<1,000	9.85 ± 5.48	0.82 (0.72–0.94)	<0.01	0.79 (0.69–0.90)	<0.01
≥1,000 and <3,000	9.00 ± 5.49				
≥3,000 and <5,000	8.16 ± 5.79				
≥5,000	8.51 ± 5.42				
**University located in a first-class city**					
Yes	9.23 ± 5.70	0.98 (0.76–1.26)	0.86	Not entered into the model	
No	9.02 ± 5.47			
**University involvement in the national “double first-class” strategic plan**					
Yes	8.95 ± 5.50	0.89 (0.69–1.14)	0.34	Not entered into the model	
No	9.44 ± 5.57			
**Research type**					
Basic scientific research	9.63 ± 5.61	0.92 (0.82–1.03)	0.15	Not entered into the model	
Clinical research	8.36 ± 5.39			
Both	9.25 ± 5.47			
Uncertain	8.79 ± 5.57			
**Duration of research work (hours, per day)**					
0–2	8.38 ± 5.37	1.26 (1.17–1.36)	<0.01	1.32 (1.21–1.44)	<0.01
3–5	7.81 ± 5.01				
6–8	9.00 ± 5.70				
9–11	9.85 ± 5.42				
≥12	11.35 ± 5.60				
**Number of participated research projects**					
0	8.93 ± 5.53	1.03 (0.92–1.14)	0.65	Not entered into the model	
1	9.00 ± 5.39			
2	9.11 ± 5.61			
3	9.43 ± 5.76			
≥4	10.12 ± 6.26			
**Participant's mentor having an administrative post**					
Yes	9.36 ± 5.23	1.06 (0.76–1.48)	0.73	Not entered into the model	
No	9.03 ± 5.56			
**Participant's mentor having and academic post**					
Yes	8.69 ± 5.28	0.90 (0.63–1.29)	0.55	Not entered into the model	
No	9.11 ± 5.55			
**Participant's mentor having projects funded by the NSFC**					
Yes	9.02 ± 5.52	1.03 (0.82–1.31)	0.80	Not entered into the model	
No	9.17 ± 5.53			
**Participants having research director**					
Yes	8.64 ± 5.39	1.42 (1.14–1.78)	<0.01	1.41 (1.12–1.77)	<0.01
No	9.79 ± 5.67				
**Number of published papers**					
0	9.18 ± 5.66	0.98 (0.90–1.06)	0.56	Not entered into the model	
1	8.91 ± 5.18			
2	8.44 ± 5.36			
3	8.96 ± 5.33			
4	9.17 ± 4.29			
≥5	8.93 ± 5.75			
**Total impact factors of published SCI papers**					
0	9.03 ± 5.57	1.06 (0.95–1.19)	0.28	Not entered into the model	
>0 and ≤ 3	8.19 ± 4.98			
>3 and ≤ 6	10.64 ± 5.51			
>6 and ≤ 10	9.05 ± 5.55			
>10	8.33 ± 5.13			
**Previous clinical diagnosis of anxiety or depression**					
Yes	14.19 ± 5.41	5.17 (3.07–8.72)	<0.01	5.38 (3.18–9.11)	<0.01
No	8.80 ± 5.40				

*SD, standard deviation; GAD-7, Patient Generalized Anxiety Disorder 7; OR, odds ratio; CI, confidence interval; Ph.D., Doctor of Philosophy Degree; RMB, Renminbi; NSFC, National Natural Science Foundation of China; SCI, Science Citation Index*.

### Risk Factors for Predicting Depression

The multivariate analysis indicated that department (surgery, gynecology, and obstetrics) (OR = 0.95, 95% CI: 0.91–0.99, *p* = 0.02), clinical medical students (OR = 1.38, 95% CI: 1.09–1.75, *p* = 0.01), longer research duration (hours per day) (OR = 1.27, 95% CI: 1.17–1.39, *p* < 0.01), no research directors (OR = 1.53, 95% CI: 1.22–1.92, *p* < 0.01), and previous clinical diagnosis of anxiety or depression (OR = 6.95, 95% CI: 4.01–12.04, *p* < 0.01) were significantly related to higher depression scores (Table 5), while participants' university involvement in the national “double first-class” strategic plan (OR = 0.73, 95% CI: 0.53–0.94, *p* = 0.02) and higher disposable income per month (OR = 0.84, 95% CI: 0.74–0.96, *p* = 0.01) were significantly associated with lower depression scores.

**Table 5:** The univariate and multivariate analysis of variables for predicting depression in Chinese medical graduate students.

**Characteristics**	**PHQ-9** **(mean ± SD)**	**Simple logistic regression**		**Multiple logistic regression**	
		**OR (95% CI)**	***P***	**OR (95% CI)**	***P***
**Age (years)**					
<25	8.85 ± 6.01	1.09 (0.92–1.28)	0.32	Not entered into the model	
≥25 and <30	9.38 ± 6.68			
≥30	9.45 ± 6.27			
**Pursued degree**					
Master's degree	9.06 ± 6.37	1.14 (0.90–1.44)	0.27	Not entered into the model	
PhD	9.37 ± 6.19			
Postdoctoral	10.08 ± 7.44			
**Grade**					
First year	8.50 ± 6.00	1.21 (1.08–1.37)	<0.01	Not entered into the model	
Second year	9.14 ± 6.42			
Third year	10.11 ± 6.60			
Fourth year	10.05 ± 6.94			
Deferment	11.47 ± 6.53			
**Department**					
Basic medicine	8.39 ± 6.41	0.96 (0.92–1.00)	0.03	0.95 (0.91–0.99)	0.02
Internal medicine	9.44 ± 6.10				
Surgery	10.00 ± 6.59				
Stomatology	8.24 ± 5.89				
Gynecology and obstetrics	10.52 ± 7.19				
Ophthalmology	8.00 ± 6.92				
Anesthesiology	9.40 ± 6.40				
Others	8.46 ± 6.05				
**Professional type**					
Academic	9.06 ± 6.37	1.07 (0.87–1.33)	0.52	1.38 (1.09–1.75)	<0.01
Clinical	9.24 ± 6.29				
**Marital status**					
Single	8.97 ± 6.19	1.03 (0.91–1.16)	0.66	Not entered into the model	
Dating	9.41 ± 6.59			
Married without children	9.18 ± 6.26			
Married with children	9.00 ± 6.13			
**Number of children**					
0	9.16 ± 6.35	1.07 (0.78–1.47)	0.69	Not entered into the model	
1	8.49 ± 6.10			
≥2	11.47 ± 5.84			
**Disposable income (RMB, month)**					
<1,000	9.87 ± 6.70	0.88 (0.77–1.00)	0.05	0.84 (0.74–0.96)	0.01
≥1,000 and <3,000	9.06 ± 6.13				
≥3,000 and <5,000	8.40 ± 6.13				
≥5,000	8.69 ± 6.79				
**University located in a first-class city**					
Yes	9.20 ± 6.29	1.02 (0.79–1.32)	0.88	Not entered into the model	
No	9.13 ± 6.35			
**University involvement in the national “double first-class” strategic plan**					
Yes	8.90 ± 6.23	0.80 (0.62–1.02)	0.08	0.73 (0.53–0.94)	0.02
No	9.93 ± 6.59				
**Research type**					
Basic scientific research	9.79 ± 6.61	0.93 (0.83–1.05)	0.23	Not entered into the model	
Clinical research	8.34 ± 6.01			
Both	9.34 ± 6.17			
Uncertain	8.79 ± 6.65			
**Duration of research work (hours, per day)**					
0–2	8.54 ± 5.93	1.21 (1.12–1.30)	<0.01	1.27 (1.17–1.39)	<0.01
3–5	7.72 ± 5.77				
6–8	9.23 ± 6.55				
9–11	9.77 ± 6.17				
≥12	11.46 ± 7.03				
**Number of participated research projects**					
0	9.14 ± 6.51	1.00 (0.90–1.11)	0.91	Not entered into the model	
1	9.05 ± 6.02			
2	9.49 ± 6.58			
3	8.60 ± 6.46			
≥4	9.74 ± 7.32			
**Participant's mentor having an administrative post**					
Yes	10.00 ± 6.60	1.25 (0.90–1.74)	0.19	Not entered into the model	
No	9.03 ± 6.29			
**Participant's mentor having an academic post**					
Yes	8.22 ± 5.75	0.84 (0.59–1.20)	0.33	Not entered into the model	
No	9.25 ± 6.39			
**Participant's mentor having projects funded by the NSFC**					
Yes	9.04 ± 6.24	1.02 (0.81–1.29)	0.85	Not entered into the model	
No	9.37 ± 6.55			
**Participants having research directors**					
Yes	8.64 ± 6.26	1.52 (1.21–1.90)	<0.01	1.53 (1.22–1.92)	<0.01
No	10.00 ± 6.38				
**Number of published papers**					
0	9.29 ± 6.45	0.98 (0.90–1.07)	0.62	Not entered into the model	
1	9.08 ± 6.45			
2	8.21 ± 5.68			
3	9.40 ± 5.70			
4	7.61 ± 4.85			
≥5	9.17 ± 6.38			
**Total impact factors of published papers**					
0	9.11 ± 6.40	1.03 (0.92–1.15)	0.62	Not entered into the model	
>0 and ≤ 3	8.44 ± 6.05			
>3 and ≤ 6	10.39 ± 6.25			
>6 and ≤ 10	9.08 ± 6.22			
>10	8.67 ± 5.41			
**Previous clinical diagnosis of anxiety or depression**					
Yes	16.38 ± 7.40	6.41 (3.73–11.01)	<0.01	6.95 (4.01–12.04)	<0.01
No	8.77 ± 6.05				

*SD, standard deviation; GAD-7, Patient Generalized Anxiety Disorder 7; OR, odds ratio; CI, confidence interval; Ph.D., Doctor of Philosophy Degree; RMB, Renminbi; NSFC, National Natural Science Foundation of China; SCI, Science Citation Index*.

### Performance Evaluation of Significant Characteristics

**Figure 5:**
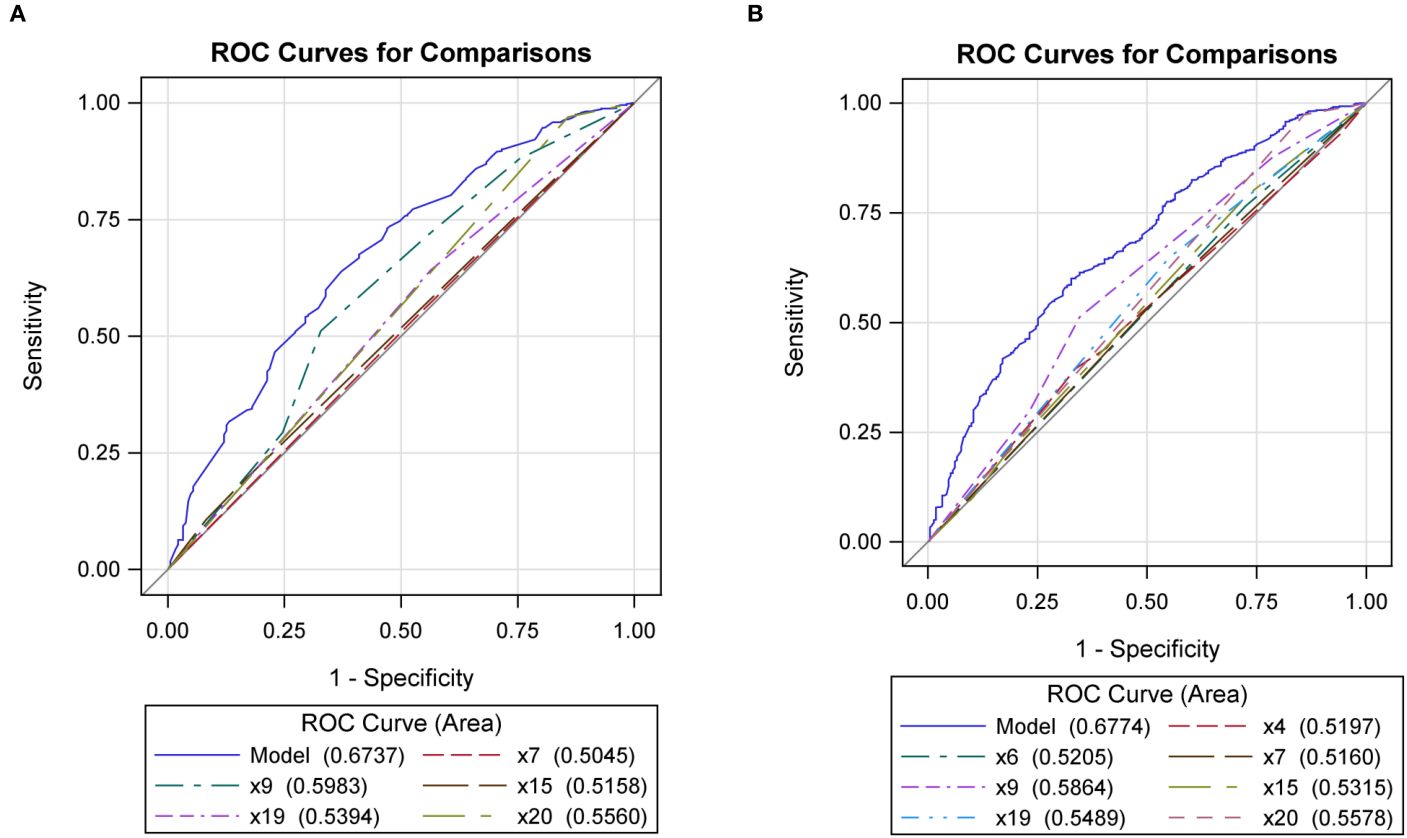
Receiver operation curves (ROC) for anxiety **(A)** and depression **(B)** among significant variables (*x*4 represents department; *x*6 represents university involvement in the national “double first-class” strategic plan; *x*7 represents the professional type; *x*9 represents research work time; *x*15 represents disposable income per month; *x*19 represents having a research director; *x*20 represents a previous clinical diagnosis of anxiety or depression).

An ROC curve was drawn (Figure 5A) to evaluate the significant characteristics associated with anxiety. The AUROC curve was 0.67 when the five significant characteristics were combined (Table 6). The correct classification rate was 83.0%, with a sensitivity of 6.6% and a specificity of 98.7%. The *p*-value from the goodness-of-fit test was 0.58.

**Table 6:** Performance evaluation of all significant characteristics associated with anxiety or depression.

**Models**	**AUROC**	**Correct classification rate**	**Sensitivity**	**Specificity**	**False positive rate**	**False negative rate**	**Goodness-of-fit test**
Anxiety	0.67	83.0%	6.6%	98.7%	50.0%	16.2%	0.58
Depression	0.68	81.2%	14.2%	97.5%	42.3%	17.6%	0.48

*AUROC, area under receiving operating characteristic*.

Figure 5B shows the ROC curve for depression. The AUROC was 0.68 with a correct classification rate of 81.2%. The sensitivity and specificity were 14.2 and 97.5%, respectively. The *p*-value from the goodness-of-fit test was 0.48.

## Discussion

The study showed that biomedical students in China suffer from severe mental health problems. Specifically, 39.02% of the biomedical students had scores indicating moderate to severe anxiety, and the scores for 37.54% of the participants indicated moderate to severe depression. These results are consistent with the other studies. Recently, a survey of 2,279 subjects from 26 countries showed that 41 and 39% of the graduate students experienced anxiety and depression, compared with 6% of the general population (21). In 2016, a meta-analysis showed a prevalence of 27.2% of depression or depressive symptoms among medical students based on samples from 167 cross-sectional studies and 16 longitudinal studies from 43 countries (22). A recently published survey of postdoctoral stress found that the severe pressure and career uncertainty seriously threatened mental health, forcing many postdoctoral students to consider abandoning their careers in science (23). In addition, the 2019 COVID-19 pandemic, as a public health emergency of international concern (24), has compounded postdoctoral pressure on career prospects (25). A cross-sectional study on the impact of the COVID-19 pandemic on psychological stress in college students in the United States showed increased stress among a majority of the participants (71.26%) during the pandemic (26).

The study is the first to show that 7 h of research work per day is the best cutoff for mental health status. In addition, at that rate of research hours, 37.04% of the biomedical students had already published at least one paper, and 25.93% had published at least one SCI paper. Although the rates of the participants having at least one published scientific paper increased with prolonged work time, the number entered a platform period, increasing very slowly after a research work duration of more than 6–7 h per day. These results might indicate that no more than 7 h of research work per day was related to the ideal mental health status and acceptable scientific achievements. When the participants worked for more than 7 h per day, their mental health status became worse, and the improvement in their scientific achievement was not evident. However, if the participants wished to publish SCI papers, prolonged research work time was more likely to be beneficial to their scientific achievements. The results highlighted the importance of a proper duration of research work time for mental health. Therefore, finding a balance between research and mental health is important for those who want to improve their academic performance while maintaining their mental health.

The results of the multiple logistic regression analysis also suggested that the research work time was significantly associated with anxiety and depression. Prolonged research work time per day increased the risk of psychological problems, possibly because, after a long period of concentration, graduate students were prone to negative emotions, especially when they lacked mental health guidance. Several other common predictors of anxiety and depression were identified in the study. Specifically, clinical medical students, those with no research directors/assistants, and having previous clinical diagnoses of anxiety or depression were associated with higher anxiety and depression scores. In this study, academic research activities were measured using the duration of academic research work. In China, policy dictates that clinical rotations are only conducted by the clinical medical students not by the academic medical students. Therefore, clinical medical students have to take clinical rotations for at least 3 years and have a limited time to complete their scientific research. Thus, it was understandable that clinical medical students suffered from more anxiety and depression due to the burdens from their clinical jobs and their scientific research tasks, in comparison with the academic medical students who were not obliged to complete a clinical training program. This finding is consistent with the study conducted by Allen et al. (27), who found that clinical doctoral students were significantly more likely to report high stress and anxiety than academic doctoral students. It was also understandable that anxiety and depression would decrease if the participants could call on directors or assistants when problems arose in scientific research. A previous clinical diagnosis of anxiety or depression was associated with the higher anxiety and depression scores, having the highest odds rates among all identified significant risk factors.

There were some protective, independent risk factors. A higher disposable income was relevant to lower anxiety and depression scores, in line with the other studies (28). Participants' university involvement in the national “double first-class” strategic plan was also significantly associated with lower depression scores. This might be because these universities are supported by more research funds and favorable national actions and policies.

According to this study, measures could be taken to alleviate the anxiety and depression of the biomedical students. First, clinical medical students could have their clinical rotation time shortened, leaving more time, perhaps at least 6 months, for their scientific research. Second, a research director/assistant should always be available to biomedical students when they encounter difficulties in their research so that problems can be solved promptly. Third, increasing the income of biomedical students would improve their mental health status, possibly due to decreased economic burdens. Fourth, biomedical students should not do more than 7 h of research work a day. This is the recommended duration because doing research work for 7 h per day was the best cutoff point for mental health and was also associated with acceptable scientific research achievements. Fifth, in some cases, low-work efficiency made the graduate students devote more time to their research, resulting in attenuated mental health. Improving work efficiency to maintain an appropriate work time might be one of the best ways to improve the mental health of the biomedical students. Therefore, we suggest that academic institutes introduce experienced professors to promote technical teaching because high-quality teaching could improve the efficiency of the biomedical students' research performance, shorten the length of their work time, and decrease the risk of psychological problems.

Compared with graduate students from other faculties, the period of education of the Chinese biomedical students is longer (29), resulting in increased economic pressure and uncertainty about the future (30). Concerns about job prospects cause increased psychological pressure. Increasing competition also forces the biomedical students to be more competitive by devoting themselves to research to improve their academic performance. This study provides a basis for further intervention study and a reference for decision-making by leaders of university departments of education and health. The department heads might need to consider the mental status of graduate students to help them complete their studies. The academic community should also take measures to build a harmonious research environment for the biomedical students. As supervisors of the biomedical students, they should pay adequate attention to students' mental health and provide psychological and economic support. For graduate students, appropriate psychological communication is conducive to relieving pressure when encountering difficulties. In other words, our research suggests that more attention should be paid to the biomedical students with signs of anxiety and/or depression. Early detection, timely supports, and proper interventions are essential for the mental wellbeing of biomedical students.

### Strengths and Limitations

To our knowledge, this study was the first to elucidate the relationship between research activities and mental health. We established that 7 h of research work a day is the best cutoff point for mental health status. Many concrete suggestions have been proposed and recommended to address mental health disorders among biomedical students. Furthermore, our findings provided a clear visual analysis of research duration, scientific research activities, and anxiety and depression scores.

However, this study has some weaknesses. First, the survey participants were biomedical students in China, and the sample representativeness might be biased because the sampling method used was not random. However, this study included a large number of participants from all over China, and the non-probability snowball sampling strategy was used. Second, although this study only collected and analyzed 20 potential risk factors, some other risk factors, such as rotation work time and gender, which could affect the students' mental health were not considered for analysis. Only two types of biomedical students were included in the study: the first were academic medical students who could do scientific research with no clinical rotation. Their only working hours were their research hours. The second type were the clinical medical students, who had to do clinical rotations for about 3 years and also complete the scientific research required for graduation. Therefore, the work hours of these students included clinical rotation hours and research hours. The study demonstrated that clinical medical students had higher anxiety and depression than the academic medical students. Therefore, the study indirectly proved that excess rotation work affected the mental health of the students. Many research studies have identified the impact of gender on the mental health of the students (31, 32). Therefore, we were not needed to investigate this variable. Third, this was a cross-sectional study, and we did not carry out longitudinal follow-ups. Thus, this study could not establish a causal relationship (33). Consequently, this study needs to be expanded in future research.

## Conclusion

Anxiety and depression are common among biomedical students. A research work duration of 7 h per day is the best cutoff for mental health, and it is associated with acceptable scientific research achievements. Not more than 7 h a day spent on research is recommended for biomedical students to maintain a balance between mental health and scientific research achievements.

## Data Availability Statement

The raw data supporting the conclusions of this article will be made available by the authors, without undue reservation.

## Ethics Statement

The study protocol complied with the Declaration of Helsinki, was approved by the Ethics Committee of the Plastic Surgery Hospital (Institute), Chinese Academy of Medical Sciences (CAMS), Peking Union Medical College (No. 2020–157), and was registered at www.chictr.org.cn (No. ChiCTR2000039574). The patients/participants provided their written informed consent to participate in this study.

## Author Contributions

YuL, SJ, and YaL: study design. BP, ML, and YaoL: data collection, analysis, and interpretation. SJ and ML: drafting of the manuscript. FG and TL: critical revision of the manuscript. All authors approved the final version for publication.

## Conflict of Interest

The authors declare that the research was conducted in the absence of any commercial or financial relationships that could be construed as a potential conflict of interest.

## Publisher's Note

All claims expressed in this article are solely those of the authors and do not necessarily represent those of their affiliated organizations, or those of the publisher, the editors and the reviewers. Any product that may be evaluated in this article, or claim that may be made by its manufacturer, is not guaranteed or endorsed by the publisher.
