# Supplementary material for: The Effects of Research Activities on Biomedical Students' Mental Health: A National Cross-Sectional Study
Source: Front Psychiatry. 2022 Mar 29;13:796697. doi: 10.3389/fpsyt.2022.796697 (PMC9004543; doi:10.3389/fpsyt.2022.796697)
Supplement: Supplementary file 1 [file Data_Sheet_1.pdf]

Supplementary Table 1 | Number of published papers in corresponding different working time groups.

Time (hours)	Students (n=1079)	Number of published papers						P [a]
		0	1	2	3	4	≧5	
0	63	55	6	0	0	1	1	
1	110	89	7	5	2	3	4	
2	135	106	18	3	2	2	4	
3	90	65	14	3	2	3	3	
4	72	50	14	4	2	0	2	
5	48	30	9	6	1	1	1	
6	80	50	11	5	8	1	5	
7	27	17	4	1	2	0	3	<0.01
8	119	74	22	12	5	3	3	
9	41	22	10	5	2	0	2	
10	132	77	22	15	7	3	8	
11	10	6	0	3	0	0	1	
12	77	42	17	7	5	4	2	
13	10	8	2	0	0	0	0	
≥14	65	33	13	8	7	2	2	

[a] indicates P values obtained from the Chi-square test.

Supplementary Table 2 | The total impact factor of published papers in corresponding different working time groups.

Time (hours)	Students (n=1079)	Total impact factor of published papers					P [a]
		0	0-3	3-6	6-10	＞10	
0	63	60	2	0	0	1	
1	110	99	8	3	0	0	
2	135	122	9	3	0	1	
3	90	74	8	7	1	0	
4	72	60	5	3	3	1	
5	48	38	5	3	1	1	
6	80	57	8	5	6	4	
7	27	20	4	0	1	2	<0.01
8	119	85	14	12	5	3	
9	41	27	6	6	2	0	
10	132	88	8	20	8	8	
11	10	6	0	2	1	1	
12	77	45	14	8	4	6	
13	10	8	0	1	1	0	
≥14	65	39	5	15	4	2	

[a] indicates P values obtained from the Chi-square test.
